# Neuropsychological analysis of anxiety and executive control of motor patterns in athletes and non-athletes

**DOI:** 10.3389/fpsyg.2024.1424152

**Published:** 2024-06-13

**Authors:** José María Caramés, Rafael E. Reigal, Verónica Morales-Sánchez, José Luis Pastrana-Brincones, M. Teresa Anguera, Antonio Hernández-Mendo

**Affiliations:** ^1^Department of Personality, Assessment and Psychological Treatment, Faculty of Psychology and Speech Therapy, University of Malaga, Málaga, Spain; ^2^Department of Social Psychology, Social Anthropology, Social Work and Social Services, University of Malaga, Málaga, Spain; ^3^School of Computer Science and Engineering, University of Málaga, Málaga, Spain; ^4^Department of Computer and Information Sciences, College of Arts and Sciences at the University of St. Thomas, Saint Paul, MN, United States; ^5^Faculty of Psychology, Institute of Neurosciences, University of Barcelona, Barcelona, Spain

**Keywords:** Finger Tapping Test, anxiety, executive functions, cognitive inhibition, sport psychology

## Abstract

**Introduction:**

Even simple tapping tasks require cognitive processes. Some variants of the Finger Tapping Test (FTT) may reveal cognitive aspects associated with frontal processing, including executive functions such as inhibition, or emotional aspects such as anxiety. A context of particular interest for the application of cognitive-motor-anxiety interactions is sports. Although athletes generally exhibit better anxiety levels, they may experience heightened anxiety before important competitions. The problem lies in determining whether the application of anxiety control techniques can be useful in pre-competition situations, given the lack of quick and easy methods to detect if an athlete is experiencing anxiety at a particular moment.

**Methods:**

This exploratory study evaluated anxiety using online versions of questionnaires (ISRA, the Competitive State Anxiety Inventory-2, and STAI) and applied a variant of the FTT to 204 participants, both athletes and non-athletes. The scores were compared and correlated.

**Results:**

Athletes exhibited lower general anxiety and greater cognitive resistance to interference (better cognitive inhibition). Non-athletes displayed a particular parameter in the FTT variant that differed from the one obtained by athletes and exhibited higher anxiety levels. In the athletes’ group only, anxiety was correlated with a specific parameter of the FTT task.

**Discussion:**

Our conclusion is that this parameter holds potential relevance in elite sports performance to detect if an athlete is experiencing anxiety. It could be of particular interest in psychological interventions in sports. Further investigation is warranted to fully explore this potential.

## 1 Introduction

The Finger Tapping Test (FTT) was developed as part of the Halstead-Reitan Neuropsychological Test Battery (HRNB) for neuropsychological evaluation (Halstead, 1947; for reviews, Allen, 2011; Schatz, 2011). The original test mainly measures motor speed as the average number of tapping events that a patient emits in 5 trials of 10 s, aimed at evaluating brain injuries (Russell et al., 1970; Allen, 2011). This original version of the task involves a high level of motor requirements, as evidenced in functional Magnetic Resonance Imaging (fMRI) studies, which showed that participants performing the FTT exhibited Blood Oxygenation Level Dependent (BOLD) activity in motor-related brain areas, such as the motor cortex, premotor cortex, supplementary motor cortex, cerebellum, and cortico-striatal-thalamic loop circuits (Turesky et al., 2018). These areas are related to movement speed, coordination, and rhythm maintenance.

However, and very interestingly, motor areas are not the only ones active. The same authors reported brain activity (meaning, significant BOLD signal) in non-motor areas, such as the inferior and middle occipital gyrus, lingual gyrus, anterior cerebellum, thalamus, supramarginal gyrus, cuneus, and left insular cortex (Turesky et al., 2018). This implies that even highly motor-related activities, such as tapping tasks, require non-motor cognitive processes to be performed (Holtzer et al., 2006; Bielak et al., 2010). Further evidence exists regarding the cognitive involvement in this task. For instance: (a) frontal lesions due to surgery, without damage in motor cortices, reduce tapping task performance (Leonard et al., 1988); (b) FTT is sensitive to some cognitive aspects, to mood states related to dementia, to certain neurodegenerative disorders (Wefel et al., 1999; Arias et al., 2012), acquired brain injury (Geldmacher and Hills, 1997), neuropsychiatric diseases (Heaton et al., 1978; Flashman et al., 1996), and to certain pharmacological substances with cognitive/psychiatric effects (Shaw et al., 1987; Swift and Tiplady, 1988; Roth and Bättig, 1991; Heishman et al., 1994); and (c) performance in FTT predicts cognitive decline (Camicioli et al., 1998).

Despite its significance, the cognitive aspects underlying the Finger Tapping Test (FTT) are not as thoroughly understood as its motor counterparts. Furthermore, its interaction with other systems, such as emotional or motivational processes including anxiety, remains less explored. While FTT use is primarily confined to motor assessment, deficient motor performance may result from impaired cognitive processing, the effects of which are manifested in the motor system. For example, white matter lesions associated with decreased speed processing may adversely affect motor skills, even though their etiology is not exclusively motor-related (Hinton et al., 2018; Andreasen et al., 2019; Rasooli et al., 2023). Hence, it is imperative to comprehend the cognitive processes involved in this task comprehensively, including their relationships with other domains. The development of new FTT variants that facilitate such investigations holds promise for advancing basic research and its application in specific contexts, such as sport psychology.

In this context, certain FTT variants modify the speed of different tapping blocks during the test to assess central versus peripheral fatigue (Arias et al., 2012; Aydin et al., 2016), or alternate sequences of normal-quick-normal-slow rhythms (González, 2001; Mendo et al., 2011). These variants have the potential to reveal cognitive challenges associated with inhibition and interactions with other neuropsychological systems related to executive functions or the proper adjustment of motor patterns. The rationale behind this lies in the fact that altering motor rhythms necessitates inhibiting previous motor patterns, cognitive flexibility to alternate between different speeds in a correct order, and executive attentional oversight of the task. In essence, changing rhythms demands executive functions associated with prefrontal brain areas (Fuster, 2001; Tirapu-Ustárroz et al., 2008a,b; Funahashi and Andreau, 2013), their projections onto limbic regions or the insular cortex (Selemon and Goldman-Rakic, 1988), and basal nuclei, primarily the caudoputamen, which have direct effects on the corticostriatal-thalamic loop (Graybiel et al., 1994; Beste et al., 2018; Florio et al., 2018), directly implicated in the correct execution of FTT (Turesky et al., 2018).

On the other hand, neurobiological circuits associated with anxiety are among the neuropsychological systems that may interact with cognitive aspects underlying FTT when changes in rhythms are required (for reviews on the neuroanatomy of anxiety, see Etkin, 2010; Schmidt et al., 2018; Goossen et al., 2019). Briefly, although amygdala is the main anxiogenic nucleus within the limbic system (Etkin and Wager, 2007), other brain regions, such as the insula and cingulate cortices, also become co-activated during uncertain situations or states of anticipatory anxiety (Sarinopoulos et al., 2010). Simultaneously, dorsolateral and orbitofrontal prefrontal cortices show decreased activity, particularly in the presence of emotional conflict (Comte et al., 2015). Impaired prefrontal attentional control has been directly linked to anxiety in some studies (Bishop, 2009). All these regions have been associated with executive functions and/or are implicated in their integration with motor patterns (Selemon and Goldman-Rakic, 1988; Graybiel et al., 1994; Fuster, 2001; Funahashi and Andreau, 2013; Beste et al., 2018) suggesting that they could be nodes of particular interest for understanding the interactions among cognitive, motor, and emotional (anxiety) domains. If so, they could be elucidated by varying classic FTT conditions, such as altering rhythmic patterns (as done previously in González, 2001; Mendo et al., 2011).

If validated, the application of this FTT variant could be particularly relevant in the context of sport psychology. Generally, athletes exhibit lower levels of anxiety overall (excluding pre-competitive anxiety states, as discussed below) compared to non-athletes (Tilindiene et al., 2014). This may be attributed to higher stress exposure during sport practice or their training to develop coping strategies for such situations (Rice et al., 2019). Regardless, this adaptation to stressful circumstances enhances their control over anxiety, mitigating the effects of environmental demands even in other aspects of life (Correia and Rosado, 2019).

However, despite the general reduction in anxiety levels due to sport practice, most athletes experience a heightened state of anxiety before important competitions, known as pre-competitive anxiety. This state is driven by uncertainty, anticipatory anxiety, and emotional conflict prior to competitions. It is observed in athletes but not in control groups (those who do not engage in competitive sports and thus lack pre-competitive exposure), complicating comparative studies. This anxiety state is likely associated with increased activity in the insular and cingulate cortices (Sarinopoulos et al., 2010) and hypoactivity in prefrontal regions, including the dorsolateral and orbitofrontal cortices (Bishop, 2009; Comte et al., 2015). These brain regions are also crucial for various aspects of executive functions required for different FTT variants.

Given their shared neural substrates, FTT variant tasks that require executive functions (such as resistance to interference) could serve as indirect assessments of anxiety. These tasks are valuable, quick, easy to interpret, and inexpensive. In elite sports, such assessments could potentially make the difference between winning and losing by addressing performance errors preemptively through the application of anxiety-control techniques, if necessary, before competition.

This research aims to delineate patterns of anxiety and motor pattern alternation (requiring executive functions such as cognitive inhibition and resistance to interference) in athletic populations, with the expectation of identifying statistically distinct ranges compared to non-athletic populations. Additionally, it endeavors to establish statistically significant relationships between performance on the Finger Tapping Test and self-reported anxiety levels using various questionnaires.

## 2 Materials and methods

### 2.1 Participants

A total of 204 young adults participated in this research (55.69% female; 44.31% male), with ages ranging from 21 to 28 years (*M* = 22.14; SD = 1.57). G*Power software (v.3.1.9.7; Heinrich-Heine-Universität Düsseldorf, Düsseldorf, Germany) was used to calculate the sample size. For the tests used in the study, with a type I and II error probability of 5%, a confidence level of 95% and statistical power of 0.95, the minimum required sample size was 168. Participants were from Andalusia, Spain. 46.08% of the sample practiced sports (Athletes group), between one to five days a week, while 53.92% do not have physical activity (No-Athletes group). There were no statistically significant age differences between groups (Athletes: *M* = 21.91; SD = 1.37; No-Athletes: *M* = 22.33; SD = 1.70) (*Z* = −1.59, *p* > 0.05). The athletes group was heterogeneous, including people who compete at local, regional and national levels, in different sports, including individual, adversarial and team sports (e.g., athletics, judo, tennis, basketball, football, among others). All athletes had eight or more years of sports experience. Inclusion criteria were to be aged between 20 and 30 years and completing all items on the questionaries. Exclusion criteria included being outside the specified age range, incomplete questionaries responses, and having any physical or psychological condition that could compromise the study. The range of 20–30 years was selected because frontal lobe maturation (and thus execute functions) is complete at that stage (Gogtay et al., 2004).

### 2.2 Measurements and instruments

(a) Inventory of Anxiety Situations and Responses (ISRA, Miguel-Tobal & Cano-Vindel, 1994). The ISRA has an S-R (situations-responses) format, made up of 22 situations and 24 anxiety responses. The 22 situations have been grouped into four areas or specific anxiety traits: evaluation situations, interpersonal situations, phobic situations and everyday life situations. The 24 responses are grouped into three subscales that evaluate the three response systems: cognitive, physiological and motor. The internal consistency values (Cronbach’s Alpha) for this research were as follows: cognitive = 0.81, physiological = 0.78 and motor = 0.83.

(b) Competitive State Anxiety Inventory–2. This questionnaire was proposed by Martens et al. (1990) and assesses competitive anxiety and self-confidence, using 27 items and three factors: cognitive anxiety, somatic anxiety and self-confidence. In this investigation, the Spanish version developed by Capdevila (1997) was used. The results were evaluated using a Likert scale from one (almost never) to five (almost always). The internal consistency values (Cronbach’s Alpha) for this research were as follows: cognitive anxiety = 0.73, somatic anxiety = 0.85 and self-confidence = 0.90. This questionnaire was done only by Athletes group.

(c) State Trait Anxiety Inventory (STAI, Spielberger et al., 1970). This questionnaire allows evaluating state and trait anxiety. It consists of 40 items and two factors: state and trait anxiety. Scores in each can range from 0 to 60 points. Each item is answered based on 4 levels 0, 1, 2, and 3. The internal consistency values (Cronbach’s Alpha) for this research were as follows: state anxiety = 0.76 and trait anxiety = 0.74.

(d) Finger Tapping Test v. 1.0.39^1^ (Mendo et al., 2011). It consists of an online variant of the classic task originally developed by Halstead (1947), applied on MenPas platform. While the original task primarily aimed to evaluate motor control, FTT-based variants could modify different parameters related to associated theoretical aspects. For example, alterations in the speed of tapping blocks may reveal central versus peripheral fatigue (Arias et al., 2012; Aydin et al., 2016). In this variant, alternate sequences of normal-quick-normal-slow rhythms were modified based on previous findings (González, 2001; Mendo et al., 2011). Briefly, the task requires clicking on a button that appears on the screen for 10 s with 4 different rhythms, in a specific order: first, normal speed (labelled as A); second, high speed (quick); third, normal speed again (labelled as B); fourth, slow speed. Frequency of clicking and latency between pulses are recorded for each velocity, although only frequency was taken into account for this study. Despite normal speed being repeated in the first and third positions, there is an interference speed (quick) between them. Thus, by subtracting A – B execution parameters, we could reveal this putative interference. The closer to 0 the A - B value is, the less the interference of the quick phase affects the “normal” speed B. Resistance to interference has been linked to executive functions, such as cognitive inhibition and flexibility (Friedman and Miyake, 2004; Diamond, 2013); and the failure to inhibit distractors has been associated with anxiety (Bishop, 2009).

### 2.3 Procedure

Initial contact with participants and data Research Topic for this exploratory study were conducted using the MenPas psychosocial evaluation platform (see Footnote 1) between January and June 2023. Voluntary cooperation was requested for participation in the research, and informed consent was obtained. The sample selection method was non-probabilistic, using convenience and snowball sampling techniques. Participants had access to the researchers’ contact details and could request information about the study. They were informed that they needed to complete various questionnaires and perform the Finger Tapping Test variant task, which took approximately 45 min to complete. After explaining the purpose of the study and ensuring anonymity and confidentiality in the handling of the data, the evaluation was conducted. Throughout the study, ethical principles outlined in the Helsinki Declaration (World Medical Association, 2013) were adhered to. The study received approval from the ethics committee of the University of Malaga (Spain). Data were stored securely on the platform and downloaded using a password-protected Excel sheet. Subsequently, the data were exported to the SPSS statistical package for analysis.

### 2.4 Data analysis

Descriptive and inferential analyses were performed. The normality of the data distributions was also tested by means of the Kolmogorov–Smirnov test. In addition, the reliability of the different scales used was analyzed by evaluating internal consistency (Cronbach’s alpha). To analyze the correlations between the measures considered, the Spearman coefficient was used, considering the parameters described by Evans (1996) (±0.01 to ±0.19 = very weak correlation; ±0.20 to ±0.39 = weak correlation; ±0.40 to ±0.59 = moderate correlation; ±0.60 to ±0.79 = high correlation). The Mann–Whitney was used to determine possible differences between groups, while Wilcoxon test was used to compare within group. They were applied given that Kolmorogov-Smirnov tests indicated that data did not fit with normal distributions. Besides, Cohen’s d was used to estimate the effect size of the differences [≈0.20: small, ≈0.50: medium, and ≈0.80: large (Hojat and Xu, 2004)]. The level of significance was set at α = 0.05. Likewise, the effect size was calculated using Cohen’s d. The SPSS statistical package was used for the statistical treatment of the data (SPSS Inc. v.25.0, Chicago, IL, United States).

## 3 Results

In Table 1, the means, standard deviations, skewness, and kurtosis values for the study variables are presented. Overall, the skewness and kurtosis values fall within acceptable ranges. However, Kolmogorov–Smirnov tests indicate significant results (*p* < 0.05) for most dimensions, both for the total sample and depending on the sport practiced. This suggests a deviation from normal distribution for the dataset, so non-parametric statistic was used when appropriated.

**Table 1:** Descriptive data.

	Total				Athletes				Non-Athletes			
	M	SD	S	K	M	SD	S	K	M	SD	S	K
ISRA - Cognitive	132.89	67.07	0.20	−0.71	114.46	60.93	0.32	−0.61	148.65	68.32	0.03	−0.75
ISRA - Physiological	82.90	60.32	0.99	0.37	67.35	52.05	1.80	3.97	96.20	63.86	0.52	−0.64
ISRA - Motor	84.33	66.24	1.02	0.29	68.41	66.47	1.60	1.90	97.94	63.23	0.70	−0.22
CSAI2 - Cognitive anxiety	–	–	–	–	30.55	3.76	0.36	0.48	–	–	–	–
CSAI2 - Somatic anxiety	–	–	–	–	26.00	8.51	−0.14	−0.75	–	–	–	–
CSAI2 – Self-confidence	–	–	-	-	33.51	7.06	−0.22	−0.24	–	–	–	–
STAI – State anxiety	17.36	10.21	0.78	0.20	13.54	9.23	1.10	1.46	20.62	9.90	0.74	−0.22
STAI – Trait Anxiety	20.17	10.11	0.03	−0.66	17.12	10.87	0.25	−0.89	22.78	8.63	0.23	−0.56
FTT - Normal rhythm A	14.11	7.98	1.94	4.13	15.81	9.97	1.51	1.64	12.66	5.40	1.71	4.85
FTT - Fast rhythm	56.63	7.10	−0.55	4.09	57.50	6.24	−1.36	6.09	55.88	7.71	−0.09	3.53
FTT - Normal rhythm B	14.69	7.24	1.54	3.76	15.54	8.41	1.67	3.58	13.95	6.01	0.81	0.55
FTT - Slow rhythm	7.84	3.63	1.46	3.31	8.13	4.07	1.15	1.85	7.59	3.20	1.83	5.74
FTT - Normal rhythm A-B	−0.57	3.85	1.10	5.46	0.27	4.57	1.53	3.52	−1.29	2.93	−1.30	3.47

Descriptive statistics for the total sample and based on sports practice in measures of ISRA, CSAI-2, STAI, and Finger Tapping Test. M, Mean; SD, Standard Deviation; S, Skewness; K, Kurtosis.

Table 2 presents differences based on the type of sports activity undertaken. It is evident that, across all comparisons, the group engaged in sports activities (Athletes) exhibits lower scores on anxiety scales. Specifically, individuals participating in sports activities demonstrate lower levels of state and trait anxiety, as well as reduced cognitive, physiological, and motor anxiety responses (*p* < 0.001). However, while a distinct pattern is observed between the groups of athletes and non-athletes in the Finger Tapping Test, with athletes displaying a higher tapping frequency and shorter execution time, these differences are not statistically significant in most cases (*p* > 0.05). However, statistically significant differences were found between the tapping frequencies in normal rhythm A minus normal rhythm B (*p* < 0.05).

**Table 2:** Comparisons between athletes and non-athletes.

	U Mann–Whitney	Z	p	Cohen ‘d	CI (95%)
ISRA - Cognitive	3656.00	−3.60	< 0.001	0.55	[0.27, 0.83]
ISRA - Physiological	3791.00	−3.28	< 0.001	0.49	[0.21, 0.77]
ISRA - Motor	3292.00	−4.47	< 0.001	0.46	[0.18, 0.74]
CSAI2 - Cognitive anxiety	–	–	–	–	–
CSAI2 - Somatic anxiety	–	–	–	–	–
CSAI2 – Self-confidence	–	–	–	–	–
STAI – State anxiety	2933.50	−5.33	< 0.001	0.74	[0.45, 1.02]
STAI – Trait anxiety	3570.00	−3.81	< 0.001	0.58	[0.30, 0.86]
FTT - Normal rhythm A	4528.50	−1.53	0.126	–	–
FTT - Fast rhythm	4461.00	−1.69	0.091	–	–
FTT - Normal rhythm B	4875.50	−0.70	0.483	–	–
FTT - Slow rhythm	4856.00	−0.75	0.452	–	–
FTT - Normal rhythm A-B	4333.00	−2.01	< 0.05	0.41	[0.14, 0.69]

Group comparisons for the ISRA, CSAI-2, STAI, and Finger Tapping Test measures.

Additionally, the Wilcoxon test (not included in the table) indicated that there were no statistically significant differences between the pulsation frequency of the normal rhythm phase A and B in the athlete sample [Z = −0.75; *p* > 0.05; Cohen’s d = −0.02, 95% CI (−0.43, 0.37)], but there were in the total sample [Z = −3.68; *p* < 0.001; Cohen’s d = 0.08, 95% CI (−0.20, 0.35)], and non-athletes [Z = −4.34; *p* < 0.001; Cohen’s d = 0.23, 95% CI (−0.15, 0.60)].

Table 3 displays the correlation analyses conducted. For the total sample, statistically significant inverse correlations were observed between taps frequency in Normal Rhythm A and the cognitive and motor scales of the ISRA, as well as between Normal Rhythm A and B and trait anxiety. Among athletes, statistically significant inverse correlations were found between the frequency of taps in Normal Rhythm A and B with the cognitive scale of the ISRA, self-confidence, and trait anxiety. Furthermore, differences between Normal Rhythm A and B correlated significantly and positively with somatic anxiety and state anxiety. Conversely, the non-athlete group showed no statistically significant relationships with the ISRA and STAI factors.

**Table 3:** Correlations matrix between FTT, anxiety measures as function of the group.

		FTT - Normal rhythm A	FTT - Fast rhythm	FTT - Normal rhythm B	FTT - Slow rhythm	FTT - Normal rhythm A-B
Total	ISRA - Cognitive	−0.14^*^	0.04	−0.13	−0.08	−0.06
	ISRA - Physiological	−0.04	−0.04	−0.01	−0.02	−0.05
	ISRA - Motor	−0.16*	−0.11	−0.10	0.01	−0.04
	CSAI2 - Cognitive anxiety	–	–	–	–	–
	CSAI2 - Somatic anxiety	–	–	–	–	–
	CSAI2 – Self-confidence	–	-	–	–	–
	STAI – State anxiety	−0.07	−0.08	−0.09	−0.01	0.10
	STAI – Trait anxiety	−0.17^*^	0.02	−0.18^*^	−0.04	−0.02
Athletes	ISRA - Cognitive	−0.21^*^	−0.01	−0.22^*^	−0.08	−0.07
	ISRA - Physiological	−0.05	0.04	0.01	0.08	−0.10
	ISRA - Motor	−0.14	0.02	−0.09	0.02	−0.09
	CSAI2 - Cognitive anxiety	0.01	0.03	0.05	0.01	0.04
	CSAI2 - Somatic anxiety	−0.07	−0.05	−0.12	−0.05	0.22^*^
	CSAI2 – Self-confidence	0.25*	0.01	0.23^*^	0.08	−0.06
	STAI – State anxiety	−0.04	0.02	−0.14	0.08	0.25^*^
	STAI – Trait anxiety	−0.20*	−0.10	−0.21^*^	−0.02	−0.05
Non-Athletes	ISRA - cognitive	0.01	0.07	−0.01	0.01	0.05
	ISRA - Physiological	0.01	−0.08	0.01	−0.09	0.03
	ISRA - Motor	−0.09	0.02	−0.09	−0.04	−0.02
	CSAI2 - Cognitive anxiety	–	–	–	–	–
	CSAI2 - Somatic anxiety	–	–	–	–	–
	CSAI2 – Self-confidence	–	–	–	–	–
	STAI – State anxiety	−0.03	−0.07	−0.04	−0.06	−0.04
	STAI – Trait anxiety	−0.10	−0.06	−0.14	−0.07	0.03

Spearman correlation analysis between ISRA, CSAI-2, STAI measures and the Finger Tapping Test (total sample by sports participation).

## 4 Discussion

The aim of this research was to observe differences in patterns of anxiety and executive function capacity to alternate between different rhythms of motor patterns among athletic and non-athletic populations. Additionally, it sought to identify statistically significant relationships between performance on the Finger Tapping Test (FTT) and levels of anxiety assessed. Initially, statistically significant differences were observed between athletes and non-athletes in cognitive, physiological, and motor anxiety responses measured with the ISRA, as well as in state and trait anxiety assessed with the STAI. Consequently, the non-athletic sample exhibited higher levels of anxiety across these measures. Regarding participants’ performance in the FTT task, there were no statistical differences between groups in any rhythm (normal A, quick, normal B, or slow). Importantly, Wilcoxon analysis revealed that when subtracted A – B, the Athlete group tended to pulse an equal number of clicks in B as in A, indicating no interference of the quick pattern on B. However, the No-Athletes group expressed more pulses in B than in A, which may reflect less cognitive inhibition to suppress the intermediate quick motor pattern. Thus, the only differences in FTT parameters between groups are evident in pulsation frequencies in Normal Rhythms A minus B, with a more stable execution observed in athletes. Furthermore, no statistically significant relationships were found between FTT performance and anxiety measures in the non-athletic sample, while some significant relationships were noted between FTT execution rhythms and certain anxiety measures in the athletic sample (Table 3).

The results suggest that athletes generally experience lower levels of anxiety compared to non-athletes, consistent with findings from previous studies (Stubbs et al., 2018; Reigal et al., 2021), while the no athletic group exhibited higher scores in terms of both anxiety state and trait (Tilindiene et al., 2014). This is in line with research indicating that physical exercise and sports participation can mitigate anxiety symptoms, enhance stress-coping abilities, and provide individuals with resources and strategies to better navigate environmental pressures (Tilindiene et al., 2014; Dale et al., 2019). Engaging in physical activity serves as a distraction, facilitating relaxation processes and reducing the impact of daily stress and anxiety (Kandola and Stubbs, 2020). Furthermore, exercise promotes the synthesis of endorphins and serotonin, enhancing emotional regulation and mood (Alizadeh Pahlavani, 2024). Furthermore, sports practice enhances the functioning of neural circuits, including those situated in prefrontal areas, which play a key role in inhibitory control (Diamond, 2013; Guiney and Machado, 2013). These circuits are directly associated with emotional regulation through connections with the limbic system and other regions mentioned in the introduction, thereby contributing to improved stress and anxiety management (Gunther et al., 2022; Shanok et al., 2022).

Taking together, the data suggest that athletes not only exhibit lower overall anxiety levels but also display more stable A-B punctuations in normal patterns, with minimal interference from the quick pattern (tending towards zero). Conversely, non-athletes tend to demonstrate more interference from the quick pattern (as their tapping in normal rhythm B is higher than in normal rhythm A), and exhibit higher anxiety scores. The correlation matrix reveals weak correlations between FTT and anxiety scores, primarily observed in athletes. It may be attributed to the higher absolute FTT scores in athletes before subtracting A - B. Additionally, correlations are only evident in athletes, not in non-athletes, possibly due to the highly heterogeneous nature of the athlete sample, reflected in the higher standard deviation of FTT scores in this group.

Importantly, this exploratory study provides initial (to our knowledge) evidence suggesting the potential use of the FTT variant employed in the study as a tool to elucidate certain executive functions related to anxiety in athletes vs. non athletes population. However, further research is warranted to address the limitations of the current study (see below). While FTT task, or its variants, have been widely utilized in various domain such as dementias, acquired brain injury (Leonard et al., 1988; Arias et al., 2012), basic neuroscientific research (Bielak et al., 2010; Turesky et al., 2018), sport psychology (González, 2001; Mendo et al., 2011), and others (see introduction), it was done separately. The findings from this exploratory investigation suggest a convergence of disciplines, indicating the potential for future studies to explore novel applications of the FTT task. These areas of interest could include its application in sports, the psychological processes underlying anxiety, or its neurobiological basis.

The underlying rationale is that engagement in sports enhances frontal brain functioning, particularly inhibitory control (Diamond, 2013; Guiney and Machado, 2013), which is functionally linked with various psychological and neurobiological processes, including anxiety (Bishop, 2009). Having a quick, easy-to-use, and inexpensive tool such as this FTT variant could greatly aid in detecting these interactions. While the data presented here are preliminary, sharing them could be valuable given their potential implications. Future research could further investigate which aspects of sports, or which sports, potentiate these interactions (e.g., is it the physical activity itself or the social aspects of team sports?); determine the magnitude of A-B deviation necessary to indicate sensitivity to this effect, and explore other related research questions.

This research presents several limitations. Firstly, the athlete sample is highly heterogeneous, with participants practicing sports between one and 5 days a week, spanning a broad range of levels of engagement. A more refined categorization based on frequency of practice and level of competition would provide clearer insights into the relationship between sports participation and anxiety. Secondly, the study did not control for the level of sports practice, resulting in a sample that included athletes from various competitive levels, ranging from local to national competitions. This lack of control may introduce bias into the data analysis. However, due to the sample size limitations, further subgroup analyses were not feasible, and thus, the conclusions should be considered exploratory and serve as a starting point for more comprehensive investigations in the future. Thirdly, the Finger Tapping Test was administered only once, potentially introducing variability due to factors such as motivation and concentration. To mitigate this, future studies could incorporate multiple trials of the test to obtain more reliable measurements and minimize biases arising from comprehension difficulties, familiarization with the task, or attentional fluctuations during performance. Additionally, it would be valuable to stratify participants based on age and gender and conduct multilevel analyses to assess the potential influence of these sociodemographic variables on the study outcomes.

This research holds significant applied value. The FTT variant emerges as a valuable tool due to its simplicity, efficiency, and affordability in uncovering cognitive functions necessary for alternating between speed pattern, and its overlapping with other cognitive and emotional functions. It is particularly relevant in the context of sports practice among athletes and non-athletes alike. Notably, the parameter of A – B subtraction in normal rhythm execution within the FTT demonstrates considerable utility, highlighting distinctions between athletic and non-athletic populations. Among athletes, this parameter correlates with higher levels of somatic and state anxiety, indicating a potential link between inhibitory control and anxiety management. Also, in athletes tend to be 0, while non-athletes tend to be negative. While further analysis is warranted, preliminary findings suggest, as main conclusion of this work, that athletes tend to exhibit less interference from rapid patterns on normal speed execution in the FTT variant, indicative of enhanced inhibitory control and lower anxiety levels. It is evidenced specifically in the A-B scores, which is something explicit to allow sport psychologist interventions targeting precompetitive anxiety, wherein deviations from a trend towards 0 in A – B scores could signal the need for anxiety management techniques. Nevertheless, these conclusions remain tentative, underscoring the necessity for additional research to corroborate and expand upon these initial observations.

## Data availability statement

The raw data supporting the conclusions of this article will be made available by the authors, without undue reservation.

## Ethics statement

The studies involving humans were approved by Ethical committee of the University of Malaga. The studies were conducted in accordance with the local legislation and institutional requirements. The participants provided their written informed consent to participate in this study.

## Author contributions

JC: Data curation, Formal analysis, Writing – original draft, Writing – review & editing. RR: Data curation, Formal analysis, Writing – original draft, Writing – review & editing. VM-S: Conceptualization, Supervision, Writing – review & editing. JP-B: Writing – review & editing. MA: Conceptualization, Supervision, Writing – review & editing. AH-M: Conceptualization, Supervision, Writing – review & editing.

## Footnotes

^1^ www.menpas.com
